# Alanine Scanning Mutagenesis of the MEDI4893 (Suvratoxumab) Epitope Reduces Alpha Toxin Lytic Activity *In Vitro* and Staphylococcus aureus Fitness in Infection Models

**DOI:** 10.1128/AAC.01033-18

**Published:** 2018-10-24

**Authors:** C. Tkaczyk, E. Semenova, Y. Y. Shi, K. Rosenthal, V. Oganesyan, P. Warrener, C. K. Stover, B. R. Sellman

**Affiliations:** a MedImmune, Gaithersburg, Maryland, USA

**Keywords:** Staphylococcus aureus, alpha toxin, epitope, monoclonal antibodies

## Abstract

Alpha toxin (AT) is a cytolytic pore-forming toxin that plays a key role in Staphylococcus aureus pathogenesis; consequently, extensive research was undertaken to understand the AT mechanism of action and its utility as a target for novel prophylaxis and treatment strategies against S. aureus infections. MEDI4893 (suvratoxumab) is a human anti-AT IgG1 monoclonal antibody (MAb) that targets AT and is currently in phase 2 clinical development. As shown previously, the MEDI4893-binding epitope on AT is comprised of the highly conserved amino acid regions 177 to 200 and 261 to 271, suggesting these amino acids are important for AT function. To test this hypothesis and gain insight into the effect of mutations in the epitope on AT neutralization by MEDI4893, nine MEDI4893 contact residues in AT were individually mutated to alanine. Consistent with our hypothesis, 8 out of 9 mutants exhibited >2-fold loss in lytic activity resulting from a defect in cell binding and pore formation. MEDI4893 binding affinity was reduced >2-fold (2- to 27-fold) for 7 out of 9 mutants, and no binding was detected for the W187A mutant. MEDI4893 effectively neutralized all of the lytic mutants *in vitro* and *in vivo*. When the defective mutants were introduced into an S. aureus clinical isolate, the mutant-expressing strains exhibited less severe disease in mouse models and were effectively neutralized by MEDI4893. These results indicate the MEDI4893 epitope is highly conserved due in part to its role in AT pore formation and bacterial fitness, thereby decreasing the likelihood for the emergence of MAb-resistant variants.

## Abstract (precis)

Alpha toxin (AT) is a cytolytic pore-forming toxin that plays a key role in Staphylococcus aureus pathogenesis; consequently, extensive research was undertaken to understand the AT mechanism of action and its utility as a target for novel prophylaxis and treatment strategies against S. aureus infections. MEDI4893 (suvratoxumab) is a human anti-AT IgG1 monoclonal antibody (MAb) that targets AT and is currently in phase 2 clinical development.

## INTRODUCTION

The spread of antibiotic resistance, along with a better understanding of the adverse effects broad-spectrum antibacterial therapy has on the beneficial microbiome, have led to the exploration of alternative approaches to antibacterial therapy, including pathogen-specific monoclonal antibodies (MAbs) to prevent or treat serious bacterial infections (1, 2). MEDI4893 (suvratoxumab) is a high-affinity anti-Staphylococcus aureus alpha toxin (AT) MAb that is currently in phase 2 clinical development for the prevention of S. aureus pneumonia in mechanically ventilated patients colonized with S. aureus in the lower respiratory tract (3). Previous studies demonstrated that AT acts as a key virulence factor in numerous preclinical disease models, including dermonecrosis, lethal bacteremia, and pneumonia (4–7). There is also evidence that AT is important in human disease, as high AT expression levels by colonizing isolates was linked to progression to pneumonia in ventilated patients (8), and low serum anti-AT IgG levels correlate with increased risk for recurrent skin infections in children (9). AT exerts its toxic effects by forming pores in target cell membranes, leading to cell lysis at higher toxin levels (10). It also has effects at sublytic levels, resulting in disruption of epithelial and endothelial tight-cell junctions, a damaging hyperinflammatory response in the lung, and evasion of killing by host innate immune cells (11–13). Alpha toxin is secreted as a soluble monomer that binds a disintegrin and metalloprotease 10, ADAM10, on cell membranes, oligomerizes into a heptameric ring, and undergoes a conformational change resulting in transmembrane pore formation in host cells, such as monocytes, lymphocytes, platelets, and endothelial and epithelial cells (10, 14).

Active and passive immunization strategies targeting AT have been reported to reduce disease severity in skin and soft-tissue infections, lethal bacteremia, and pneumonia (4, 5, 15–19). Specifically, MEDI4893*, a non-YTE version of MEDI4893, has been shown to reduce disease severity in multiple animal models (13, 17, 20) and to exhibit synergy when administered in adjunctive therapy with standard-of-care antibiotics (15, 21, 22). MEDI4893 binds with high affinity to a discontinuous epitope on AT (amino acids [aa] 177 to 200 and 261 to 271) and inhibits pore formation by blocking toxin binding to target cell membranes (20, 23). Recent studies of diverse S. aureus clinical isolate collections (∼1,250 total) demonstrated that the AT gene, *hla*, is carried by a majority (>99.5%) of isolates, and 58 different AT sequence variants were identified (24–26). In these collections, the MEDI4893 epitope was highly conserved, with only 19 isolates having mutations in the epitope. We hypothesized that such a high degree of conservation results from the amino acids in the MEDI4893 epitope playing a key function in AT lytic activity.

To better understand the role of these amino acids in the cytolytic mechanism of AT and gain insight into the effect of mutations in the AT MEDI4893-binding epitope, the residues on AT that contact MEDI4893 were mutated to alanine. The results from this study indicate the amino acids in the MEDI4893 epitope are important for toxin function and bacterial virulence and that the MAb is capable of neutralizing toxin molecules with mutations in its binding epitope. Taken together, our observations imply a low potential for emergence of AT variants resistant to neutralization by MEDI4893.

## RESULTS

### Cytolytic activity of AT alanine mutants.

**FIG 1:**
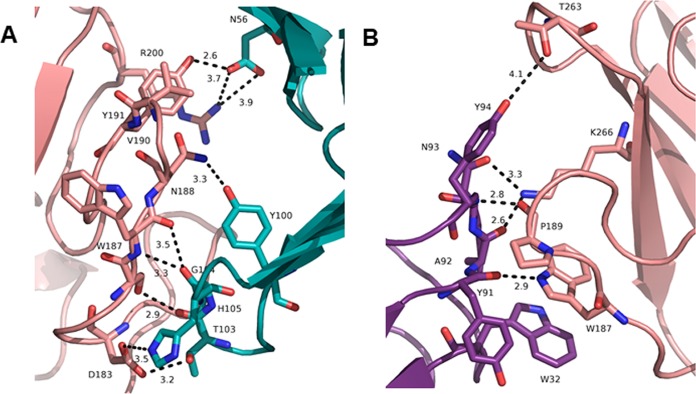
Interface between MEDI4893 Fab HC (green) and AT (pink) (A) and MEDI4893 Fab LC (purple) and AT (pink) (B). Both chains of the Fab interact with AT and create hydrogen bonds (dotted lines). Residue W187 interacts with the heavy chain (HC) through hydrogen bonds and with W32 on the light chain (LC) by π-π stacking interaction.

**FIG 2:**
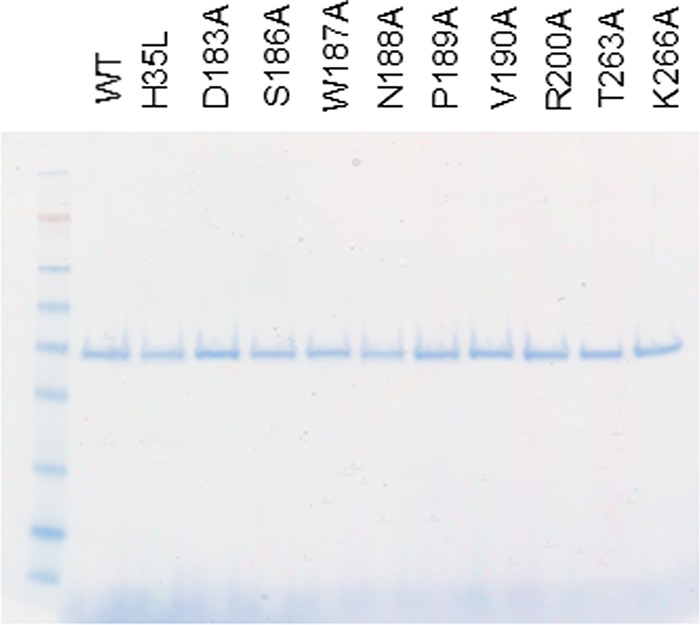
WT-AT and mutant purity. Gel Coomassie staining of all proteins (0.5 μg/well) is shown.

**FIG 3:**
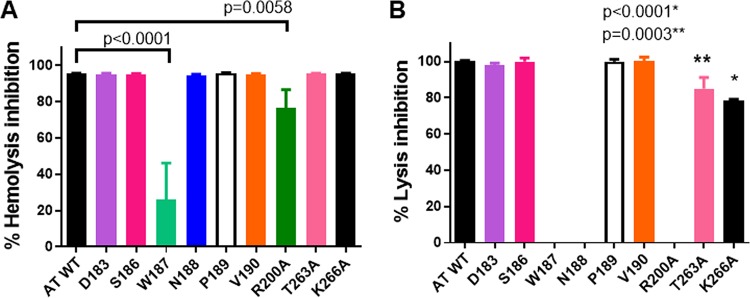
MEDI4893 neutralization *in vitro* on rabbit RBC hemolysis (A) and A549 lysis (B). WT or mutant AT were mixed with MEDI4893 serial dilutions with RBC (0.1 μg/ml) (A) or with A549 cells (20 μg/ml) (B). Percent hemolysis or cell lysis inhibition was calculated as 100 × [100 − (OD of AT plus MAb)/(OD of AT alone)]. Data were analyzed with one-way ANOVA followed by a Dunnett's multiple comparison. Results were considered statistically significant at a *P* value of <0.05. Data are representative of three independent experiments at an AT/MEDI4893 ratio of 1:2.

The crystal structure of the MEDI4893 antigen-binding fragment (Fab) bound to AT revealed a discontinuous epitope spanning amino acids (aa) 177 to 200 and 261 to 271, with direct molecular contacts on the toxin at residues D183, S186, W187, N188, P189, V190, R200, T263, and K266 (Fig. 1A and B) (23). These residues were highly conserved among ∼1,250 diverse S. aureus clinical isolates (24–26). Alanine scanning mutagenesis of these 9 contact residues was conducted to determine their role in AT function and to gain insight into the effect these mutations have on MEDI4893 neutralizing activity. Each of the 9 mutants was expressed as a full-length 33-kDa protein from S. aureus and purified from the culture supernatant by ion-exchange chromatography (Fig. 2). Cytolytic activity of AT alanine mutants was first examined on rabbit red blood cells and the A549 human lung epithelial cell line (Table 1; see also Fig. S1 in the supplemental material). As shown in Table 1 and Fig. S1B, W187A, N188A, and R200A mutants exhibited little or no cytolytic activity on A549 cells. All of the mutants, with the exception of P189A and S186A, exhibited significant loss in either hemolytic or lytic activity compared to that of wild-type AT (WT-AT) (Table 1). When MEDI4893 was incubated with either the WT or mutant toxins (MAb:AT molar ratio of 2:1) prior to the assays, the MAb exhibited similar neutralizing activity (≥95%) against all mutants in the hemolytic assay, with the exception of R200A and W187A, against which the MAb neutralized 80% and 22% of activity, respectively (Fig. 3A). MEDI4893 neutralized ≥75% of the cytolytic activity of D183A, S186A, P189A, V190A, T263A, and K266A on A549 cells (Fig. 3B). W187A, N188A, and R200A were not tested in the A549 lysis assay because of their greatly diminished lytic activity. These results indicate that amino acids in the MEDI4893 epitope are essential for AT function and that the MAb neutralizes epitope variants *in vitro*.

**TABLE 1:** AT mutant lytic activity on rabbit RBC and human A549 cell line^*a*^

WT or mutant	Activity for:			
	A549		RBC	
	LD_50_ (μg/ml)	*P* value (WT vs mutant)	LD_50_ (ng/ml)	*P* value (WT vs mutant)
WT	5.12		12.12	
D183A	12.9	<0.0001*	24.21	0.0357*
S186A	6.5	0.4039	19.71	0.2361
W187A	>20	<0.0001*	38.58	<0.0001*
N188A	>20	<0.0001*	48.87	<0.0001*
P189A	5.8	0.9413	11.14	>0.9999
V190A	12.9	<0.0001*	50.79	<0.0001*
R200A	>20	<0.0001*	197.4	<0.0001*
T263A	10.8	0.0112*	13.4	0.997
K266A	10	<0.0001*	13.61	0.9997

a Rabbit RBC or the A549 human cell line was incubated with AT at the doses indicated in Fig. S1. The 50% lethal dose (LD_50_), corresponding to the [AT] required for 50% lysis, was calculated. Statistical difference for LD_50_ between WT-AT and mutants was considered statistically significant at *P* < 0.05 and is indicated with an asterisk.

### MEDI4893* reduces AT mutant-induced dermonecrosis.

**FIG 4:**
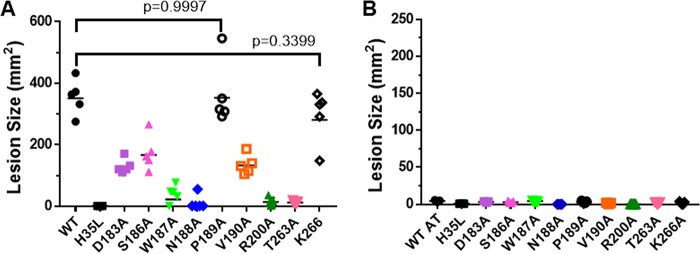
MEDI4893* inhibits purified toxin-induced dermonecrosis. BALB/c mice (*n* = 5) were immunized intraperitoneally with 10 mpk c-IgG (A) or MEDI4893* (B) and injected intradermally with WT or mutant AT (1 μg). AT_H35L_ (H35L) was included as a negative control. Dermonecrotic lesions were measured 24 h postinfection. Data were analyzed for panel A using one-way ANOVA followed by Dunnett's multiple comparison. All mutant toxins, except P189A (*P* = 0.9997) and K266A (*P* = 0.3399), resulted in lesions with results that were statistically different from those for the WT (*P* < 0.0001). Data are representative of 3 independent experiments.

AT is a key virulence factor in S. aureus skin and soft-tissue infections (5, 6, 27, 28), and intradermal (i.d.) injection of purified toxin results in dermonecrotic lesions in mice (29). To determine if the *in vitro* lytic activity translated into a similar pattern of activity *in vivo*, the capacity of each alanine mutant to induce dermonecrosis in mice was assessed. WT or mutant toxins were injected i.d. into BALB/c mice (*n* = 5), and dermonecrotic lesion sizes were recorded 24 h postinjection. Injections of either P189A or K266A mutant resulted in lesion sizes similar to those of WT toxin 24 h postinjection, whereas D183A, S186A, and V190A formed lesions significantly smaller than those of WT-AT. W187A, N188A, R200A, and T263A formed little or no detectable lesions (Fig. 4A and Fig. S2). Results were similar on day 7, except for delayed dermonecrotic lesion formation by D183A, N188A, and T263A (Fig. S2). Consistent with its *in vitro* neutralization activity, passive administration of MEDI4893* (10 mg/kg [mpk]) 24 h prior to toxin injection resulted in complete inhibition of lesion formation induced by WT-AT and all dermonecrotic AT mutants (Fig. 4B). These results indicate the *in vitro* lysis activity remains consistent *in vivo* and provides evidence that MEDI4893 effectively neutralizes *in vivo* AT mutants with amino acid changes in its binding epitope.

### AT mutations reduce pore formation by decreasing cell binding.

**FIG 5:**
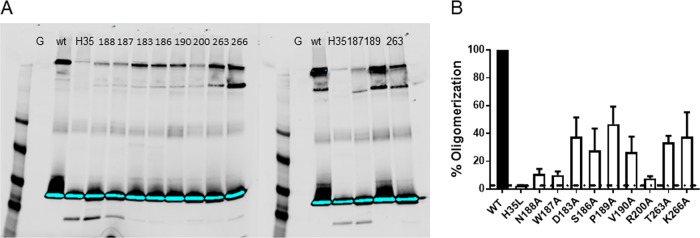
AT mutant heptamer formation. The alanine mutants were incubated with erythrocyte ghosts at 37°C. (A) Samples were solubilized in SDS-PAGE, and heptamer formation was detected by Western blot analysis. The WT and H35L-AT were used as positive and negative controls. The blot is representative of three independent experiments. (B) Percent oligomerization was calculated from mean band intensities on three separate blots. Heptamers formed by WT-AT are considered 100% oligomerization. Statistical differences between WT and AT mutants were calculated with a one-way ANOVA followed by a Dunnett's multiple comparison. All *P* values were <0.0001 and considered statistically significant.

AT lyses cells in a multistep process, whereby AT monomers bind ADAM10 on cell membranes and then oligomerize into a heptameric ring and insert into the membrane to form an SDS-resistant transmembrane pore (30). To determine if the lysis-defective mutants formed SDS-resistant heptamers in cell membranes, the alanine mutants were incubated with freshly prepared erythrocyte ghost membranes and heptamer formation was measured (31) by Western blot analysis, as previously described (20). Heptamer formation was measured by densitometry, and percent heptamer formation was calculated for each mutant relative to that of the WT (Fig. 5). The oligomerization-deficient mutant H35L was included as a negative control. Consistent with the cell lysis assays, N188A, W187A, and R200A exhibited the greatest loss in activity, whereas the other mutants lost 50 to 70% of heptamer formation in this assay. These results confirm that the amino acids in AT-MEDI4893 contact residues are essential for AT pore formation.

**FIG 6:**
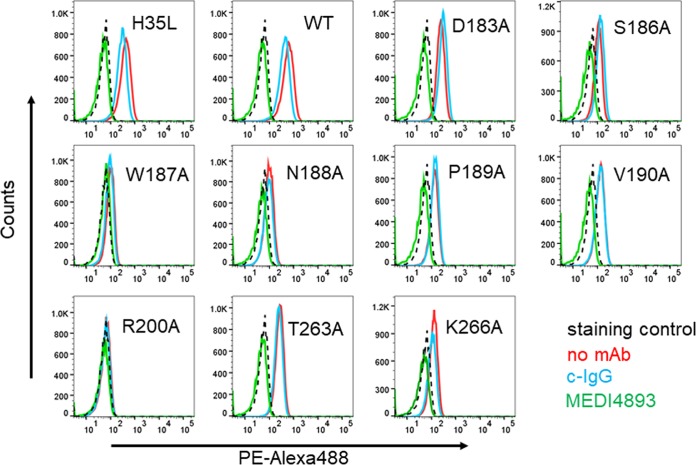
MEDI4893 inhibits AT binding to A549 cell surface. Biotin-conjugated WT or mutant AT was incubated with A549 cells in the presence of MEDI4893 (green) or c-IgG (blue). A549 cells were also incubated with toxin alone (red). The background consisted of A549 cells alone (black). AT binding was measured by cytofluorimetry after addition of phycoerythrin-Alexa 488.

Walker and Bayley previously reported that amino acid R200 is important for AT binding to cell membranes (31). To determine if the other epitope residues were also important for cell binding, the mutant toxins were biotinylated and binding to A549 cells was measured by flow cytometry (23) and compared with that of WT-AT and the oligomerization-defective mutant H35L (Fig. 6 and Table S1). Similar to the A549 lysis results, AT mutants D183A, P189A, V190A, T263A, and K266A exhibited reduced cell binding relative to that of WT-AT or H35L, whereas no binding was detected with the mutants (W187A, N188A, or R200A) most defective for A549 lysis (Fig. 6 and Table 1), indicating the residues comprising the MEDI4893 epitope are important for cell binding. Consistent with the MEDI4893 neutralization of AT mutant-mediated lysis (Fig. 3), the MAb effectively blocked detectable cell binding by all mutants (Fig. 6).

### MEDI4893 binding affinity to AT mutants.

The results described above indicated MEDI4893 effectively neutralized the lytic alanine mutants *in vitro* and *in vivo*. To further characterize the interaction of MEDI4893 with the epitope mutants, MAb association (*k*_on_) and dissociation (*k*_off_) constants for each mutant were measured by surface plasmon resonance, and the affinity constant (*K_D_*) for each variant was calculated. Although the MAb exhibited a modest drop in *K_D_* to D183A, S186A, V190A, and T263A, it retained a subnanomolar *K_D_*; however, antibody binding constants to N188A, P189A, R200A, and K266A were reduced >10-fold (11- to 26-fold), and no binding to W187A was detected in the assay. These data showed a direct correlation between decreased neutralization of AT mutants in the hemolytic assay and their loss for affinity to MEDI4893 (*r* = 0.7633) (Table 2 and Fig. S3) and confirmed that W187 is critical for both MEDI4893 binding and neutralization of AT (Table 2 and Fig. 3B).

**TABLE 2:** Association and dissociation rate constants and apparent binding constants of MEDI4893 for AT alanine mutants^*a*^

Strain	*k*_on_ (M^−1^ s^−1^)	*k*_off_ (s^−1^)	*K_D_* (nM)	Fold loss
WT	1.00e+06	1.40e−04	0.14	
D183A	1.78e+06	9.08e−04	0.511	3.5
S186A	7.90e+05	1.04e−04	0.132	0
N188A	2.40e+06	5.77e−03	2.405	16
P189A	2.13e+06	3.60e−03	1.694	11
V190A	2.34e+06	6.77e−04	0.289	2
R200A	1.17e+06	3.14e−03	2.69	18
T263A	1.37e+06	2.04e−04	0.15	0
K266A	1.35e+06	5.15e−03	3.812	26

a Association (*k*_on_) and dissociation (*k*_off_) rate constants were measured using a BIAcore instrument, and the apparent binding constant (*K_D_*) was calculated as *k*_on_/*k*_off_. Data are representative of one of three separate experiments. No binding was observed for the W187A mutant.

### MEDI4893* inhibited disease caused by SF8300 strains expressing mutant AT.

**FIG 7:**
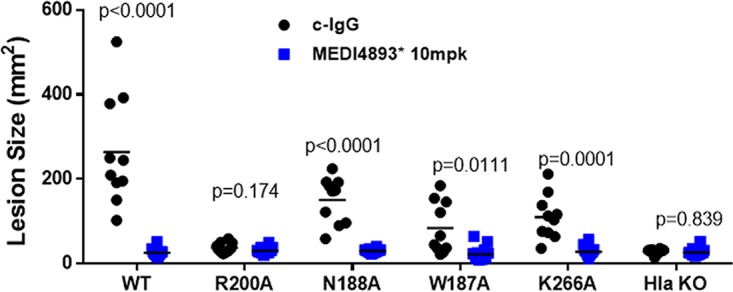
MEDI4893* inhibits USA300 knock-in mutant-induced dermonecrosis. BALB/c mice (*n* = 10) were immunized intraperitoneally with c-IgG or MEDI4893* (10 mpk) 24 h prior to intradermal infection with S. aureus SF8300 or the SF8300 *hla* knock-in mutants (5e7 CFU). Lesion sizes were measured 24 h postinfection. Differences for lesion sizes between c-IgG- and MEDI4893*-immunized mice for each strain were analyzed with a parametric *t* test. Lesion sizes for the WT versus each knock-in strain were analyzed with one-way ANOVA followed by Dunnett's multiple comparison, with *P* values of <0.0001 for R200A, W187A, and K266A and a *P* value of 0.0015 for N188A. Values for both tests were considered statistically significant at a *P* value of <0.05.

**FIG 8:**
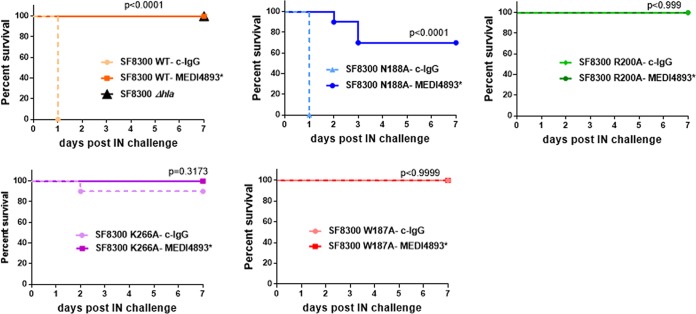
MEDI4893* inhibits USA300 knock-in mutant virulence in pneumonia. C57/B6 mice (*n* = 10) were passively immunized with MEDI4893* or c-IgG (15 mpk) and infected intranasally (IN) with 1.3e8 CFU of the WT or the Δ*hla* or *hla* knock-in mutant SF8300. Survival was monitored for 6 days postchallenge. Data were analyzed with a log-rank Mantel-Cox *t* test and considered statistically significant at a *P* value of <0.05. Data are representative of 3 independent experiments.

Although MEDI4893* fully inhibited dermonecrosis induced by i.d. injection of the nine AT mutants, four mutants (N188A, W187A, R200, and K266A) exhibited at least a 15-fold loss of binding by MEDI4893 (Table 2). To determine if the MAb prevented disease caused by S. aureus strains expressing the mutant toxins with the greatest loss in binding affinity, the alanine mutants were introduced into the USA300 CA-MRSA SF8300 chromosome by allelic exchange. Each mutant was confirmed to express equivalent toxin levels *in vitro* (Fig. S4). BALB/c mice (*n* = 10) were passively immunized with MEDI4893* (10 mpk) or an isotype control IgG (c-IgG) 24 h prior to i.d. inoculation with each strain (5e7 CFU), and lesion sizes were measured 24 h postinfection. Consistent with the *in vitro* lysis and toxin-mediated dermonecrosis results described above, the strains expressing N188A, W187A, and K266A were less virulent and formed significantly smaller lesions than infection with WT SF8300 (*P* < 0.0001), and the strain expressing the least lytic mutant, R200A, was avirulent in this model. Also, MEDI4893* prophylaxis inhibited dermonecrosis caused by the mutant-expressing strains, resulting in lesions resembling infection with an S. aureus Δ*hla* strain (Fig. 7 and Fig. S5). The AT mutant-expressing strains were similarly avirulent in the murine pneumonia model except for SF8300-N188A, which caused disease similar to that of WT SF8300. Despite an ∼16-fold drop in binding affinity for N188A, MEDI4893* prophylaxis significantly reduced death following infection with the N188A mutant strain (Fig. 8). Collectively, these data demonstrate that the AT amino acids comprising the MEDI4893-binding epitope are essential not only for AT function but also for S. aureus fitness *in vivo*, and that MEDI4893* can neutralize the toxic effects of AT even when its epitope is mutated and its binding affinity reduced.

## DISCUSSION

The current antibiotic resistance problem has been fueled by widespread use and misuse of broad-spectrum antibiotics. Coupled with a greater understanding of the adverse effects that empirical broad-spectrum antibiotic therapy has on the healthy microbiome, there has been an increasing effort to identify alternative strategies to treat infections with antibiotic-resistant pathogens. One emerging approach is the use of MAbs to either prevent or treat resistant bacterial infections (1, 32). Although several antibacterial MAb-based treatment strategies are currently in clinical testing, little information is available about resistance to these molecules. Antibacterial childhood vaccines (e.g., diphtheria, tetanus, pertussis, Haemophilus influenzae type b, and Streptococcus pneumoniae), which rely on a functional antibacterial antibody response, have been in use for years or even decades with no apparent emergence of resistance (33). This lack of resistance is likely due in part to the polyclonal antibody response generated by the vaccines and may not predict a similar outcome for an antibacterial MAb.

Unlike antibiotics, which kill bacteria and exert direct selective pressure on bacteria, MAb-based antibacterial approaches either neutralize bacterial virulence factors, promote a protective immune response, or target the bacteria for opsonophagocytic killing by host immune cells (2, 34). Consequently, the host immune system is responsible for killing the bacteria, not the MAb (35). Since MAbs do not affect bacterial growth directly, it is difficult or even impossible to study the emergence of resistance *in vitro* or *in vivo* over the time course of preclinical infection models. To begin to address the questions about circulating resistant S. aureus isolates, we conducted two studies in which the AT gene (*hla*) was sequenced from ∼1,250 clinical isolates (24–26). In these collections, 58 different AT sequence types were identified and the anti-AT MAb MEDI4893, currently in phase 2 clinical testing, effectively neutralized all lytic variants. Of these clinical isolates, only 19 encoded an AT protein with a mutation in the MEDI4893 epitope, which was effectively neutralized. During phase 1 study, no AT variants with amino acid substitutions in the MEDI4893-binding region were detected despite prolonged exposure of S. aureus strains to MEDI4893 due to its extended half-life (26). Because the MEDI4893 epitope is highly conserved and no MEDI4893-resistant mutants were observed thus far, we hypothesized that the AT amino acids in the MEDI4893-binding epitope are important for AT function.

In the current study, we mutated 9 amino acid contact residues in the MEDI4893 epitope to alanine to characterize their roles in AT lytic activity and S. aureus fitness and to gain insight into these mutations on MEDI4893 binding and neutralization. Consistent with published results indicating that residue R200, found in the epitope, was important for AT cell lysis (31), lytic activity was decreased ≥2-fold in 8 out of 9 mutants constructed here, and of these, 3 mutants had ≥10-fold-reduced levels of lytic activity on rabbit red blood cells (RBC) or the A549 lung epithelial cell line (Table 1 and Fig. S1). This loss in toxin activity *in vitro* translated into a similar loss in activity *in vivo*, resulting in reduced virulence in both skin and lung infection models. These results support the hypothesis that the MEDI4893 epitope in AT is important not only for AT lytic activity but also for bacterial fitness in skin and lung infection models. Therefore, it is not surprising that only two MEDI4893 epitope variants (N188T and V190I) were identified in the collection of ∼1,250 bloodstream and lung infection clinical isolates (24–26). Although the potential for emergence of MEDI4893 resistance appears to be low, further monitoring of AT sequence variants encoded by circulating clinical isolates is under way that will provide added information regarding AT sequence conservation and the fitness of strains expressing defective or inactive AT variants.

MEDI4893 is an affinity-optimized variant of the anti-AT MAb 2A3 (20, 21). When 2A3 was identified, its *in vitro* and *in vivo* neutralizing activity strongly correlated with MAb affinity for the toxin (20). This finding led to an affinity optimization campaign to increase affinity that would hopefully translate into increased potency and protective capacity *in vivo*. Although 2A3 was successfully optimized into MEDI4893 with ∼10-fold increased binding affinity (*K_D_* of 0.60 nM for 2A3 and ∼0.089 nM for MEDI4893), there was no improvement in neutralization activity or protection in preclinical disease models (13, 20, 29). However, the improved MEDI4893 binding affinity may have been beneficial regarding its ability to compensate for epitope mutations in AT. For example, MEDI4893 exhibited 26- and 16-fold losses in affinity for K266A and N188A, respectively, yet it retained a biologically relevant affinity (*K_D_* of 3.8 and 2.4 nM, respectively), and neutralizing activity for each mutant was sufficient to prevent disease in the dermonecrosis and pneumonia models. These results indicate that the high-affinity binding of MEDI4893 for AT helps overcome a loss in binding due to some mutations in its binding epitope. Taken together with the importance of the MEDI4893 (suvratoxumab) epitope in AT lytic activity and bacterial fitness, the high MAb affinity may provide an added hurdle for the bacteria on the pathway to develop resistance to AT neutralization by a MAb.

## MATERIALS AND METHODS

### Alpha toxin alanine mutant expression.

The wild-type *hla* gene was PCR amplified from S. aureus SF8300 (USA300) genomic DNA and cloned into a pCN-based Escherichia coli-staphylococcal shuttle vector under the control of a constitutive promoter based on the *S. aureus clpB* gene promoter (36, 37). Alanine mutant *hla* expression plasmids were prepared by cloning synthetic DNA fragments containing the mutations into the wild-type expression construct. The alanine mutant plasmids were introduced into the S. aureus RN4220Δ*hla* strain by electroporation and selected on medium containing 10 μg/ml chloramphenicol. Mutant-expressing strains were cultured overnight at 37°C in BHI (brain heart infusion) broth (Criterion, Inc.) with 10 μg/ml chloramphenicol, and AT proteins were purified from culture supernatants by cation exchange chromatography using an SP-HP column (GE Healthcare) equilibrated with 30 mM Na-acetate, pH 5.2, 20 mM NaCl, 1 mM EDTA and eluted with a linear gradient to 500 mM NaCl.

### Rabbit RBC hemolytic assay.

The rabbit red blood cell (RBC) hemolytic assay was performed as described previously (20). Briefly, wild-type AT (WT-AT) or AT mutants were serially diluted 2-fold in 50 μl starting at 10 μg/ml and incubated with 50 μl of washed RBC (Pel-Freez) for 1 h at 37°C. Plates were then centrifuged at 1,200 rpm for 3 min, and 50 μl of supernatant was transferred to new plates. Nonspecific human IgG1 R347 was used as a negative control (c-IgG) (20). The optical density at 450 nm (OD_450_) was measured with a spectrophotometer (Molecular Devices). Hemolytic activity of each AT mutant was calculated in hemolytic units per milliliter (HU/ml) as the inverse dilution corresponding to 50% hemolysis, with 10 μg/ml corresponding to 1 HU/ml.

### A549 cytotoxic assay.

Human lung epithelial cell line A549 (ATCC, Manassas, VA) was cultured at 37°C in 5% CO_2_ in Dulbecco's modified Eagle medium (DMEM; VWR International), supplemented with 10% of fetal bovine serum (Gibco) and 2 mM glutamine (Invitrogen). Cells were incubated overnight in a 96-well plate (VWR International) at 5e4 cells/well. Serial dilutions of AT mutants or WT-AT was then added to cells for 2 h at 37°C, plates were centrifuged at 2,000 rpm for 2 min, and 50 μl supernatant was transferred to a new 96-well plate. Cell lysis was measured as release of lactate dehydrogenase (LDH), using the CytoTox 96 nonradioactive assay kit (Promega) by following the manufacturer's recommendations. As a positive control, cells were lysed with 10% SDS. Background of LDH release was subtracted, and 100% of cell lysis was calculated as 100 × [(OD_490_ of cells with AT)/(OD_490_ of cells with SDS)].

### AT oligomerization on erythrocyte ghosts.

Erythrocyte ghosts were prepared as described previously (20) from rabbit blood (Pel-Freez). Briefly, 5 ml of packed RBC were washed twice in 0.9% NaCl and incubated in 90 ml of lysis buffer (5 mM Na-phosphate buffer, 1 mM EDTA, pH 7.4) overnight at 4°C under constant stirring. Ghosts were obtained after centrifugation at 15,000 × *g* for 20 min, washed 3 times with lysis buffer, washed one time in phosphate-buffered saline (PBS), and resuspended in PBS to an OD_600_ of 0.2. Heptamers were formed by incubating 5 μl ghosts with purified AT proteins (0.5 μg) and PBS in a final volume of 21 μl for 45 min at 37°C. Samples were then solubilized in 7 μl of Bolt LDS sample buffer (Invitrogen) and incubated for 5 min at room temperature, and 13 μl was run on 4 to 12% Bolt morpholinepropanesulfonic acid (MOPS) SDS gel (Life Technologies). The separated proteins were transferred to nitrocellulose membrane in Bolt transfer buffer (Invitrogen) with 10% methanol overnight at 15 V, blocked with Odyssey blocking buffer (LI-COR) for 1 h, and probed with rabbit anti-AT IgG (2 μg/ml) for 2 h at room temperature. The AT bands were detected after 1 h of incubation with IRDye 680RD-conjugated donkey anti-rabbit IgG (LI-COR) by an Odyssey fluorescence imager (LI-COR). Band intensities were calculated with Odyssey Image Studio Lite software.

### MEDI4893 affinity to AT mutants.

Kinetic rate constants (*k*_on_ and *k*_off_) for binding of the MEDI4893 to WT-AT and each mutant were measured by employing an IgG capture assay on a Biacore T200 instrument. Protein A was immobilized on a CM5 sensor chip with a final surface density of ∼2,000 resonance units (RUs). MEDI4893 was prepared at 10 nM in instrument buffer (HBS-EP buffer; 0.01 M HEPES, pH 7.4, 0.15 M NaCl, 3 mM EDTA, and 0.005% P-20), along with 2-fold serial dilutions of AT (0.048 nM to 50 nM). A sequential approach was utilized for kinetic measurements. MEDI4893 was first injected over the capture surface at a flow rate of 10 μl/min. Once the binding of the captured IgG stabilized, a single concentration of the AT protein was injected over both capture and reference surfaces at a flow rate of 50 μl/min. The resulting binding response curves yielded the association phase data. Following the injection of AT, the flow was then switched back to instrument buffer for 10 min to permit the collection of dissociation phase data, followed by a 1-min pulse of 10 mM glycine, pH 1.7, to regenerate the protein A surfaces on the chip. Binding responses from duplicate injections of each concentration of AT were recorded against anti-AT MAb MEDI4893. In addition, several buffer injections were interspersed throughout the injection series. Select buffer injections were used along with the reference cell responses to correct the raw data sets for injection artifacts and/or nonspecific binding interactions, commonly referred to as double referencing. Fully corrected binding data were then globally fit to a 1:1 binding model (BIAevaluation 4.1 software; BIAcore, Inc.) that included a term to correct for possible mass transport-limited binding. These analyses determined the kinetic rate constants *k*_on_ and *k*_off_, from which the apparent dissociation constant (*K_D_*) was calculated as *k*_off_/*k*_on_.

### Alpha toxin binding by cytofluorimetry.

AT binding to A549 cells and the inhibitory effect of MEDI4893 were measured as previously described (23), with some modifications. Briefly, AT mutants or the WT were biotinylated using the EZ-Link Sulfo-NHS-LC biotinylation kit (Thermo Fisher Scientific) by following the manufacturer's protocol. All incubations and washes were performed in fluorescence-activated cell sorting buffer (PBS, 0.5% bovine serum albumin, 0.1% Tween). AT and MEDI4893* were preincubated for 30 min at room temperature at a molar ratio of 1:5. Cells were first blocked in with human Fc blocker (eBioscience) and then incubated for 1 h at 4°C with AT alone or MEDI4893-AT mix. Following one wash, cells were then incubated for 30 min at 4°C with phycoerythrin-Alexa 488 conjugate (eBioscience). After two washes, AT binding was then quantified with an LSRII flow cytometer (BD Biosciences), and data were analyzed with FlowJo software (Tree Star, Inc., Ashland, OR).

### Chromosomal allelic exchange of *hla* alanine mutants in strain SF8300.

Alanine mutation-containing sequences were subcloned into temperature-sensitive allelic exchange vector pBD100 (Binh Diep, UCSF). Constructs were transferred from strain RN4220 into strain SF8300 by phi11 transduction. Chromosomal plasmid integrants were obtained by temperature selection and verified by PCR. Subsequent negative selection for plasmid excision was performed by plating on medium containing anhydrotetracycline. Alanine substitutions in the chromosomal *hla* gene were verified by PCR and sequencing.

### Mouse dermonecrosis.

Demonecrosis studies were conducted as previously described (20). Six-week-old female BALB/c mice (Harlan) were passively immunized with MEDI4893* (10 mpk) or c-IgG (20) and i.d. challenged 24 h later with SF8300 WT or SF8300 expressing AT alanine mutants (5e7 CFU). Dermonecrosis was also induced by i.d. injection of purified alanine mutants or the WT (diluted in cold PBS at 1 μg/50 μl). Lesion sizes were measured 24 h and 7 days after infection.

### Mouse pneumonia.

Lethal pneumonia was induced as reported previously (13). Six-week-old female C57/B6 mice (Jackson) were passively immunized with MEDI48938 (15 mpk) or c-IgG and intranasally infected 24 h later with SF8300 WT or SF8300 expressing AT alanine mutants (1.5e8 CFU in 50 μl). Animal survival was monitored over 6 days postinfection.

### Statistical analyses.

The data were analyzed using one-way analysis of variance (ANOVA) followed by Dunnett's multiple comparison, *t* tests, and log-rank Mantel-Cox test where appropriate. Welch correction was applied where variances were not similar. Differences were considered significant at a *P* value of <0.05. All tests were two sided. GraphPad Prism 7.04 was used for all statistical analyses.

### Mouse models.

All experiments were performed in accordance with institutional guidelines following experimental protocol review and approval by the Institutional Biosafety Committee (IBC) and the Institutional Animal Care and Use Committee (IACUC) at MedImmune.

## Supplementary Material

Supplemental file 1
