# Supplementary material for: Alanine Scanning Mutagenesis of the MEDI4893 (Suvratoxumab) Epitope Reduces Alpha Toxin Lytic Activity In Vitro and Staphylococcus aureus Fitness in Infection Models
Source: Antimicrob Agents Chemother. 2018 Oct 24;62(11):e01033-18. doi: 10.1128/AAC.01033-18 (PMC6201083; doi:10.1128/AAC.01033-18)
Supplement: Supplemental file 1 [file zac011187594s1.pdf]

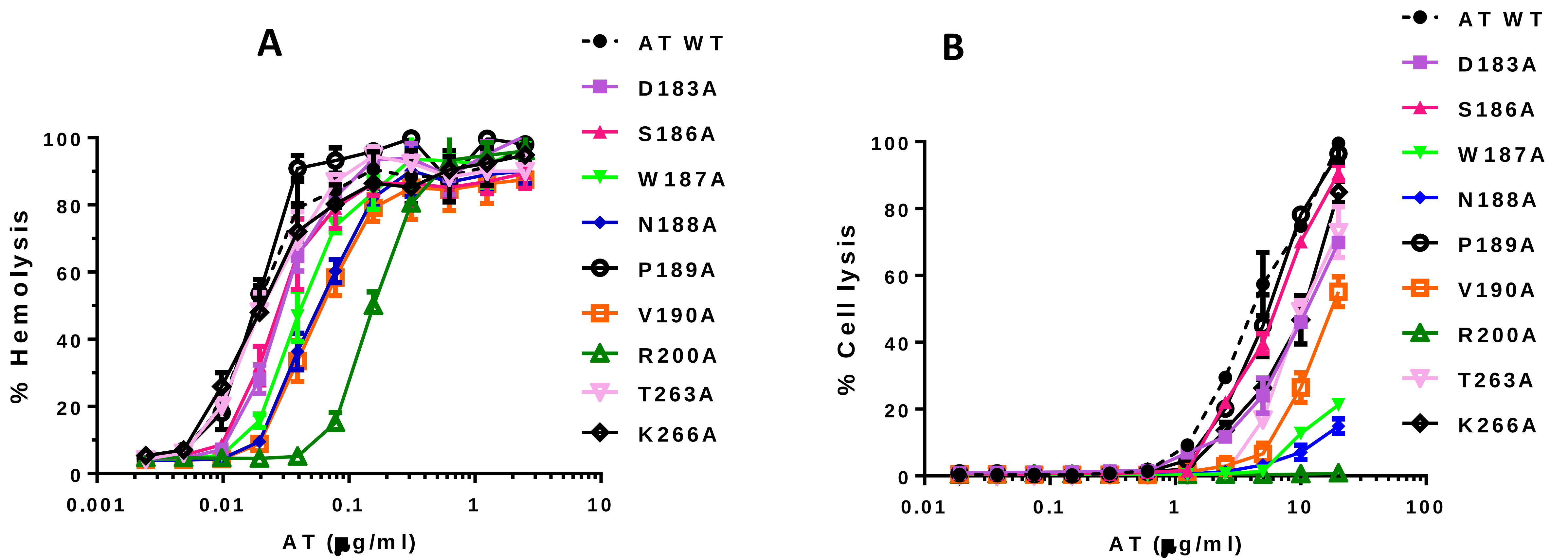

Fig. S1: AT mutant lytic activity on rabbit RBC (A) and human A549 cell line (B). (A) Washed RBCs were incubated with serial dilutions of AT WT or AT mutants (2.5 to 0.0024µg/ml). Hemolysis was measured by the amount of hemoglobin released in the supernatant, and calculated as followed: $100*[(OD_{AT})/(OD_{SDS})]$. (B) A549 were incubated with serial dilutions of AT WT or AT mutants (20 to 0.01µg/ml). LDH was measured in supernatants after two hour incubation at 37°C. % cytolysis was calculated as follows: $100*[100-(OD_{AT}) / (OD_{SDS})]$. Results are a mean of triplicates and representative of two separate experiments.

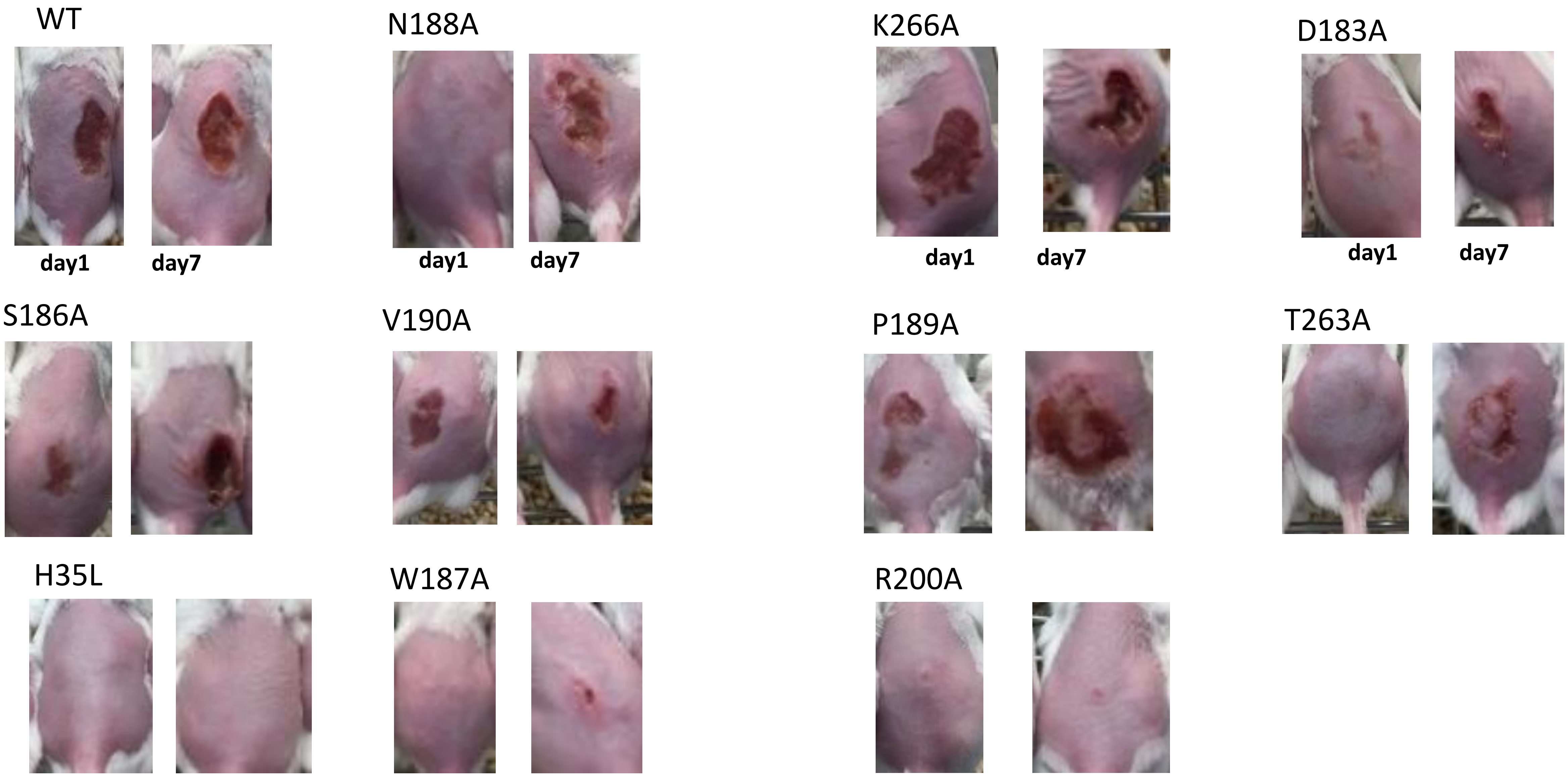

Fig. S2: Purified toxin-induced dermonecrosis. Balb/c mice (n=5) were injected intra-dermal with AT molecules (1μg). Wild type AT (WT) and AT_{H35L} (H35L) were used as positive and negative controls, respectively. Lesions were recorded at days 1 and 7 post injection. The data are representative of 3 independent experiments.

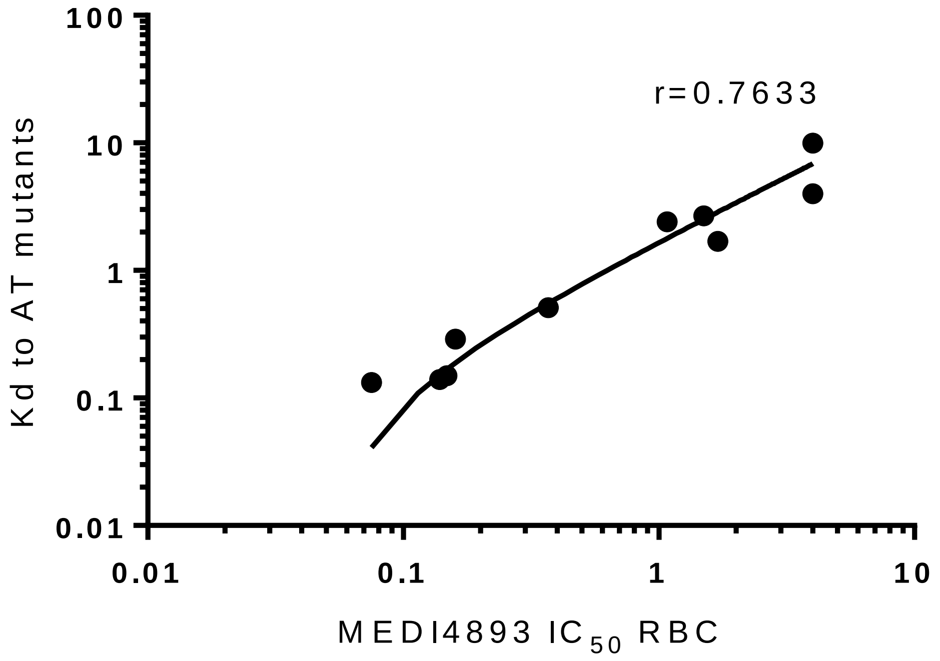

Fig. S3: Correlation between MEDI4893 neutralization in hemolysis and KD binding to mutants. Linear regression constant was measured between Constant affinity binding (Kd) and in vitro activity (IC_{50} in the hemolytic assay) for each AT mutant IC50 were graphed and linear regression constant calculated.

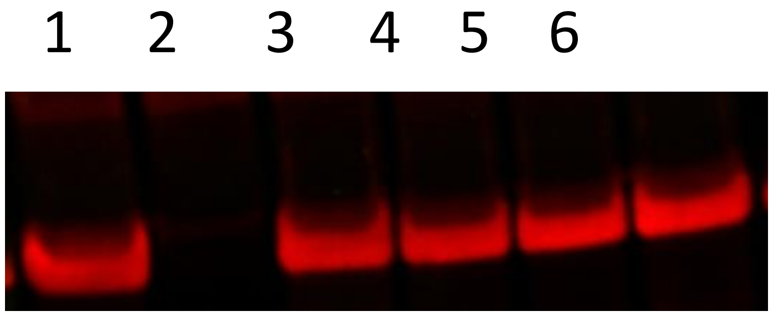

1- SF8300
2- SF8300 *ΔHla*
3- SF8300 W187A
4- SF8300 N188A
5- SF8300 R200A
6- SF8300 K266A

Fig. S4: AT expression in SF8300 knock-in mutant
Strains were cultured overnight in TSB, and AT level measured by western blot

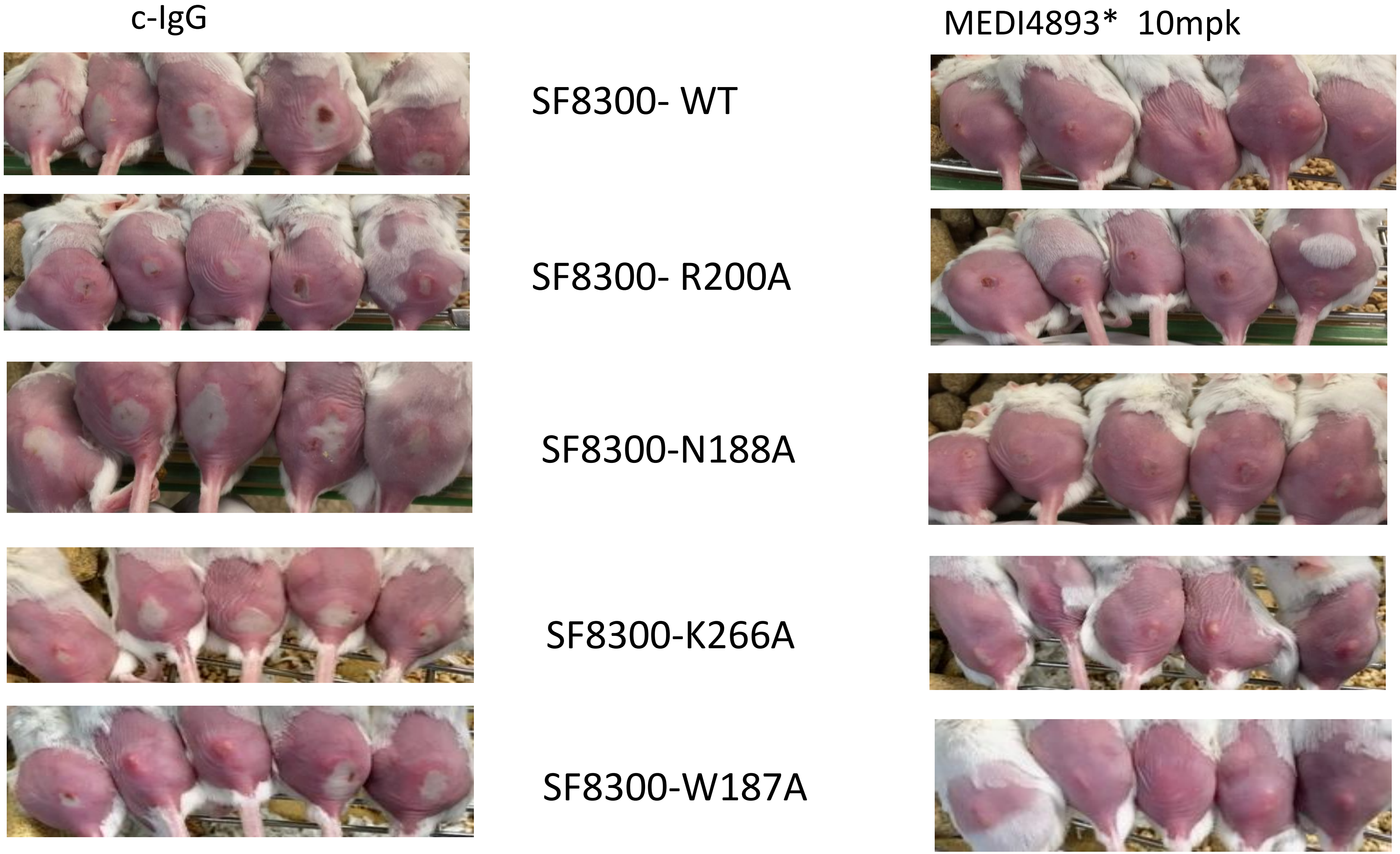

Fig. S5: MEDI4893* inhibits USA300 knock-in mutant induced-dermonecrosis. Balb/c mice (n=10) were IP immunized with c-IgG or MEDI4893* (10mpk), and ID injected with SF8300 WT or SF8300 knocking mutants (5e7cfu). Lesion sizes were photographically recorded 24h post infection. This experiment is representative of 3 independent experiments.

	H35L	WT	D183A	S186A	W187A	N188A
**no mAb**	307	415	216	62	24	28
**c-IgG**	312	470	159	40	32	21
**MEDI4893***	3	2	1	1	5	2

	P189A	V190A	R200A	T263A	K266A
**no mAb**	68	58	5	160	79
**c-IgG**	61	61	8	185	55
**MEDI4893***	2	0	4	2	0

Table S1: Median of fluorescence intensity of AT mutants and nAT on A549 human cells in presence of anti-AT mAb (MEDI4893*) or irrelevant mAb (c-IgG)
Mean for staining control has been subtracted.
